# Supplementary material for: Cross-attention PHV: Prediction of human and virus protein-protein interactions using cross-attention–based neural networks
Source: Comput Struct Biotechnol J. 2022 Oct 8;20:5564–73. doi: 10.1016/j.csbj.2022.10.012 (PMC9546503; doi:10.1016/j.csbj.2022.10.012)
Supplement: Supplementary data 1 [file mmc1.pdf]

Supplemental information

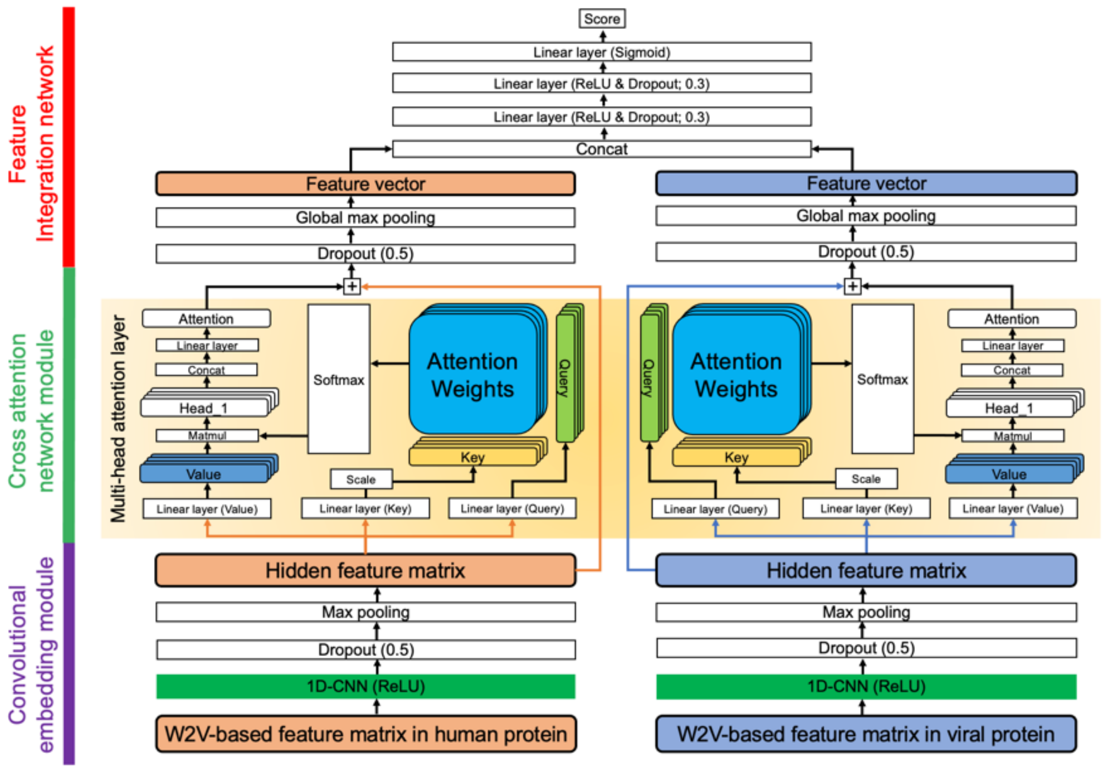

Fig. S1. Structure of the self-attention–based neural network. The network was composed of three sub-networks. The word2vec (W2V)-based feature matrices of human and virus proteins were input into the convolutional embedding module. The output matrices of human and virus proteins were then input into the separate respective multi-head attention layers. This part differs from cross-attention PHV. Finally, the feature vectors generated by the global max-pooling layer were concatenated to compute a final score through three linear layers.

Table. S1. The number of long and short proteins in each dataset.

Dataset		Short human proteins (length <= 2000)	Long human protein (length > 2000)	Short virus proteins (length <= 2000)	Long virus protein (length > 2000)
Denovo		2251	87	399	23
Human-unknown virus PPI	H1N1	19704	451	1505	99
	H3N2	19720	449	1505	99
	H5N1	19681	447	1505	99
Human-SARS-CoV-2	Balanced	14085	341	12	2
	Imbalanced	19738	454	12	2

Table. S2. Comparison of the performances of PPI prediction for short and long proteins.

Protein	Number	Positive	Negative	SN	SP	ACC	MCC	AUC	F1
Short	723	368	355	0.945	0.970	0.957	0.915	0.990	0.957
Long	127	57	70	0.944	0.949	0.946	0.892	0.961	0.940

Table. S3. Parameters of machine learning models

Classification	Parameter name	Value
DT	Criterion	gini
	Max depth of tree	None (Until the leaf reaches 1.)
	Minimum sample to split	2
RF	Criterion	gini
	Number of trees	100
	Max depth of tree	None (Until the leaf reaches 1.)
	Minimum sample to split	2
SVM	Regulation parameter (C)	1
	Kernel	rbf

Table. S4. Comparison of the cross-attention PHV with machine learning methods by the independent test on the balanced human-SARS-CoV-2 PPI dataset.

Model	Encoding	SN	SP	ACC	MCC	AUC	F1
DT	one-hot	0.786	0.756	0.771	0.542	0.771	0.774
	w2v	0.821	0.756	0.789	0.578	0.789	0.795
LR	one-hot	0.668	0.785	0.727	0.456	0.819	0.710
	w2v	0.677	0.796	0.737	0.477	0.833	0.720
RF	one-hot	0.800	0.853	0.827	0.654	0.910	0.822
	w2v	0.820	0.883	0.851	0.704	0.928	0.847
SVM	one-hot	0.750	0.788	0.769	0.538	0.858	0.764
	w2v	0.739	0.763	0.751	0.502	0.847	0.748
Cross-attention PHV		0.897	0.865	0.881	0.764	0.958	0.883

Table. S5. Comparison of the cross-attention PHV with machine learning methods by the independent test on the imbalanced human-SARS-CoV-2 PPI dataset.

Model	Encoding	SN	SP	ACC	MCC	AUC	F1
DT	one-hot	0.680	0.936	0.893	0.615	0.808	0.680
	w2v	0.724	0.940	0.904	0.658	0.832	0.716
LR	one-hot	0.299	1.00	0.883	0.512	0.821	0.460
	w2v	0.299	1.00	0.883	0.511	0.835	0.460
RF	one-hot	0.448	0.998	0.907	0.628	0.934	0.615
	w2v	0.525	0.999	0.920	0.689	0.944	0.687
SVM	one-hot	0.333	0.993	0.883	0.508	0.850	0.488
	w2v	0.427	0.989	0.895	0.569	0.887	0.576
Cross-attention PHV		0.845	0.990	0.966	0.875	0.973	0.892
